# Grapheme learning and grapheme-color synesthesia: toward a comprehensive model of grapheme-color association

**DOI:** 10.3389/fnhum.2013.00757

**Published:** 2013-11-11

**Authors:** Michiko Asano, Kazuhiko Yokosawa

**Affiliations:** ^1^Department of Environment and Information Studies, Faculty of Environment and Information Studies, Keio UniversityFujisawa, Japan; ^2^Research Fellow of the Japan Society for the Promotion of ScienceTokyo, Japan; ^3^Brain Science Institute, Tamagawa UniversityTokyo, Japan; ^4^Department of Psychology, Graduate School of Humanities and Sociology, The University of TokyoTokyo, Japan

**Keywords:** grapheme-color synesthesia, grapheme acquisition, grapheme discrimination, Japanese phonetic characters (Hiragana), language development, perceptual categorization and identification

## Abstract

Recent progress in grapheme-color synesthesia research has revealed that certain regularities, as well as individual differences, figure into grapheme-color associations. Although several factors are known to regulate grapheme-color associations, the impact of factors, including their interrelationships, on synesthesia remains unclear. We investigated determinants of synesthetic color for graphemes (characters, letters) of Hiragana, a phonetic script in the Japanese language, and the English alphabet. Results revealed that grapheme ordinality was the strongest predictor of synesthetic colors for Hiragana characters, followed by character sound, and visual shape. Ordinality and visual shapes also significantly predicted synesthetic colors for English alphabet letters, however, sounds did not. The relative impact of grapheme properties on grapheme-color associations and the differences between these two writing systems are accounted for by considering the way graphemes are processed in the brain and introduced during an individual's development. A new model is proposed which takes into account the developmental process of grapheme learning. The model provides comprehensive explanation of synesthetic grapheme-color association determination processes, including the differences across writing systems.

## Introduction

Grapheme-color synesthesia is a condition in which a visual letter or character (grapheme) induces a specific color sensation (e.g., the letter “r” may induce a concurrent sensation of “red”). Although it has been characterized as idiosyncratic, a number of regularities in the synesthetic experience have also been reported. Specifically, the factors that affect synesthetic grapheme-color correspondence are systematically associated with several grapheme properties: visual shape, sound, meaning or concepts, grapheme frequency, ordinality (positions in a grapheme sequence), and memory related to the graphemes (e.g., visual form: Brang et al., 2011; Watson et al., 2012; sound: Asano and Yokosawa, 2011, 2012; meaning or concepts: Rich et al., 2005; Asano and Yokosawa, 2012; grapheme frequency: Beeli et al., 2007; ordinality: Watson et al., 2012; memory: Witthoft and Winawer, 2006, 2013). What remains unclear is the relative impact of such various factors on synesthesia.

Some studies examined simultaneous influence of multiple grapheme properties on the same set of graphemes (Asano and Yokosawa, 2011, 2012; Watson et al., 2012). Asano and Yokosawa (2012) investigated the simultaneous influences of sound, visual shape, and meanings on synesthetic colors for Kanji characters (a logographic script used in the Japanese language) in Japanese grapheme-color synesthesia. Watson et al. (2012) also investigated the influence of sound (letter names), visual shape, ordinality, and frequency on synesthetic colors for the modern English alphabet simultaneously in English grapheme-color synesthesia. Findings from these different studies reveal that several factors are concurrently involved in grapheme-color associations. However, to date the relative size of impact among the factors has not been the subject of specific investigation.

Results of some studies suggest that the magnitude of impact of each factor differs across writing systems, which poses a difficulty for resolving the puzzle of how several factors work together. For example, synesthetic colors for the English alphabet are usually determined by graphemes, not phonemes (e.g., the “c” in “cat” and in “cite” may both elicit the same color sensation, whereas initial letters of “site” and “cite” appear to elicit different colors; Simner, 2007). It is also reported that synesthetic color similarity across the English alphabet does not correlate with letter name similarity (e.g., although the name sounds of the letter “b” and “c” are similar, they are usually associated with dissimilar colors; Watson et al., 2012). In sharp contrast to English, synesthetic colors for Japanese phonetic scripts such as Hiragana and Katakana rely on sound quality, not on visual shapes of graphemes (Asano and Yokosawa, 2011). Hiragana and Katakana represent the same set of vowels or syllables (i.e., combinations of a consonant and a vowel) although their visual forms are dissimilar. Asano and Yokosawa (2011) reported that synesthetic color choices for Hiragana characters and those for their Katakana counterparts were remarkably consistent, indicating that color selection depended on character related sounds and not visual form (effects of ordinality and frequency were not investigated in this study).

Some researchers have claimed that synesthetic associations are acquired as graphemes are learned (Rich et al., 2005; Witthoft and Winawer, 2006, 2013; Wagner and Dobkins, 2011; Asano and Yokosawa, 2012; Watson et al., 2012). The relative size of factor impact may reflect the way in which graphemes were associated with colors in the brain of a synesthete in his/her childhood, when s/he was learning graphemes. Grapheme learning consists of visual shape discrimination of graphemes and the association of graphemes with phonemes (in the case of phonetic scripts including the English alphabet) or with phonemes and meanings (in the case of logographic scripts). Watson et al. (2012) showed that letter shape and ordinality were linked to hue, and letter frequency was linked to luminance of synesthetic colors for the English alphabet in adult grapheme-color synesthetes. Based on such findings, they conjectured that associating letter shapes and identities with hue aided letter learning (categorical discrimination of letters), and that letter frequency, which varies along a continuum, mapped naturally to luminance in childhood.

Several causal factors for the association of colors with graphemes during grapheme learning have been proposed in various studies. For instance, Rich et al. (2005) raised the possibility that colors are linked to items forming conventional sequences (e.g., alphabets, numbers, days of the week, months of the year) before the acquisition of graphemes (letters, numerals); these links then are generalized to graphemes and other items that are not part of such sequences (e.g., words). Likewise, Asano and Yokosawa (2012) suggested the possibility that grapheme-based synesthesia begins as phoneme synesthesia (i.e., colors are linked to phonemes prior to grapheme acquisition). According to the observation that characters representing the same sounds tended to elicit similar colors in Japanese grapheme-color synesthesia regardless of their visual shapes, they surmised that, in some cases, the colors linked to phonemes before grapheme acquisition are subsequently generalized to graphemes via phonology. Some related evidence for early associations of simple visual shapes with colors comes from an infant study by Wagner and Dobkins (2011), in which they demonstrated that the presence of particular shapes influences color preferences in typical 2- and 3-month-olds (but not in 8-month-olds or adults). Although further research is required to clarify the relationships between such “neonatal synesthesia” and grapheme-color synesthesia, this study suggests that shape-color linkages may be formed in the brain before grapheme acquisition. External input can also be a source of synesthetic colors. Witthoft and Winawer showed that some synesthetes had startlingly similar color-grapheme pairings traceable to childhood toys containing colored letters, such as refrigerator magnets (Witthoft and Winawer, 2006, 2013). All these studies suggest the importance of developmental perspectives in achieving a complete understanding of grapheme-color association process; however, the interrelationships among the factors raised above are not still clear.

Considering previous developmental research with grapheme-color synesthesia, in the present study we propose a model that offers a comprehensive explanation of synesthetic grapheme-color association determination processes, including the differences across writing systems. Five main claims of this model are: (1) synesthetic associations are acquired as graphemes are learned. (2) Children who are learning graphemes use information from various feature domains to differentiate among graphemes. (3) Linkages between color representations and representations of features in some domains (e.g., ordinality in conventional sequences, sounds, and visual shapes) are formed in the brain before grapheme acquisition. (4) When a grapheme is learned, (one of the) color representations associated with representations of the grapheme's features is/are mapped onto the grapheme. The color for the representation of feature which makes the largest contribution in discriminating the grapheme from others is the color most likely to be ultimately associated with the grapheme. (5) The resulting synesthetic color highlights the discriminating feature(s) of the grapheme. This facilitates grapheme discrimination and in turn grapheme learning.

### Two examples: english letters and japanese characters

**Figure 1:**
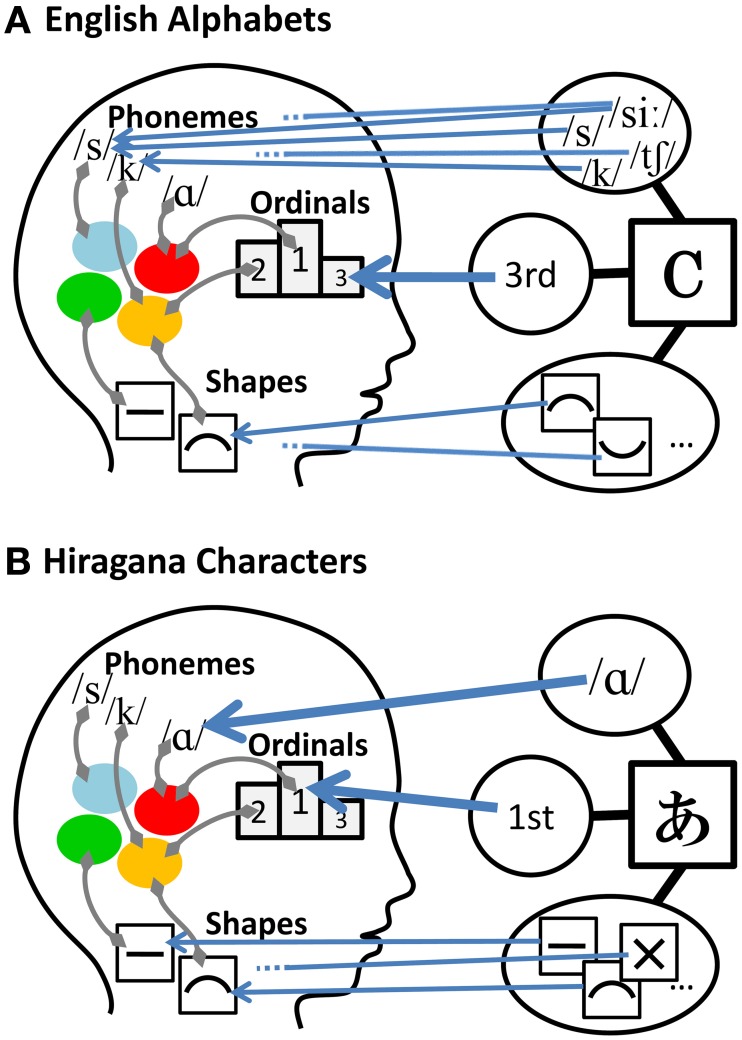
**Schematic illustrations of the comprehensive explanatory model of synesthetic grapheme-color association proposed in this study**. In the case of English alphabets **(A)** and Hiragana characters **(B)**.

Schematic illustrations of the proposed model appear in Figure 1 for each of two example language systems. First, consider a model for English alphabets, shown schematically in Figure 1A. In the English alphabet, graphemes (letters) form a sequence. The ordinality domain allows for identifying one grapheme with one ordinal representation of a character because each grapheme is assigned only one ordinal number (e.g., first, second, third).

In the visual shape domain, correspondences between graphemes and representations are rather complex compared to those in the ordinality domain. Assuming that visual shapes of graphemes are processed in units of basic distinctive visual features (cf. Gibson, 1969), one grapheme should be linked to several basic visual feature representations, because graphemes usually combine several basic visual features (except for very simple graphemes like “I” and “O,” which may be linked to only one basic visual feature representation).

For phonological domains, a highly complex relationship exists between graphemes and phonological representations in the case of the English alphabet. There are two kinds of phonological information associated with a grapheme: the name of grapheme and its pronunciation. For instance, the letter “b” has the grapheme name of “bee” and it is pronounced as /b/. In short, learning an English grapheme requires acquiring two kinds of associations of phonological information with a single grapheme. A typical English alphabet letter has several pronunciations, and the pronunciation of the letter name is usually different from any of the letter pronunciations (e.g., pronunciation of the letter “c” can be either /s/, /k/, or “ch” in “chance”; the pronunciation of the name of the letter “c” is /see/, which is different from any of the letter pronunciations). In addition to the one-to-many correspondences between graphemes andphonemes, some English phonemes are represented by several graphemes; for example, the phoneme /s/ is represented by both letters “c” and “s,” and the phoneme /z/ is represented by both “s” and “z” (i.e., many-to-one correspondences).

To summarize, there are simple (i.e., one-to-one) ordinality-grapheme correspondences, relatively complex shape-grapheme correspondences, and highly complex phoneme-grapheme correspondences in the English alphabet. When the corresponding relations between grapheme and feature representations in a certain domain are simple (e.g., when one-to-one relationship exists among graphemes and feature representations), mapping the corresponding representations (and the color associated with each of the representations) to the graphemes can provide an additional source of information available for grapheme discrimination. Complex correspondences between graphemes and representations of a given domain, on the other hand, can reduce the influence of that feature domain on grapheme-color associations. This is because such a feature domain is likely to contribute little to a differentiation among graphemes. Thus, the synesthetic color that is finally associated with a grapheme is determined. Taking these factors into account, our model predicts that ordinality should have the greatest impact on synesthetic colors for the English alphabet, followed by visual shapes. The impact of phonology on synesthesia should be very small because graphemes are arbitrarily associated with phonemes in the English alphabet. This prediction is at least partly in accordance with results of previous studies that report the influence of visual shape (e.g., Brang et al., 2011), or of ordinality and visual shape but not of sound (Watson et al., 2012; see also Simner, 2007) on grapheme-color synesthesia for the English alphabet (This is “at least” because no previous study examined the relative size of impact of these factors).

Consider a second example which involves Japanese Hiragana characters (see Figure 1B). Our model is best characterized by contrasting cases of graphemes of different writing systems with different characteristics. As noted above, Hiragana is a phonetic script that represents a set of vowels and syllables (see Table S1 in Supplementary Materials). Hiragana characters are the graphemes that most native Japanese speakers learn first (usually at around the ages of 4–6 years; see (Asano and Yokosawa, 2012), for detailed descriptions of scripts used in the Japanese language). As in the case of the English alphabet, there are simple correspondences between graphemes (characters) and ordinal representations in Hiragana characters, as each grapheme is assigned only one ordinal number. In the domain of visual shape, as in the case of the English alphabet, correspondences between graphemes and representations are somewhat complex compared to those in the ordinality domain. This is because graphemes are made of combinations of several basic visual features.

In the phonological domain, however, Hiragana characters are very different from English alphabet letters. In the case of Hiragana characters, each character has only one pronunciation and it is the same as the pronunciation of the character name (e.g., the name of the first character in the Hiragana sequence is “a” (/a/) and the character is also pronounced as /a/). Consequently, there is a one-to-one relationship between graphemes and phonemes^1^ in Hiragana characters. This reasoning leads to the prediction that phonology and ordinality are strong determinants of synesthetic colors and that the visual shapes of characters should have less impact in grapheme-color synesthesia for Hiragana characters. This prediction is partly consistent with Asano and Yokosawa (2011). In their study synesthetic colors for Hiragana characters were found to be elicited by sound qualities and not by visual shapes. Thus, the proposed model has the ability to explain the differences in determinants of grapheme-color association across writing systems.

### A new model

The present model goes beyond simply providing a theoretical backbone to existing observations. Using this model, we can predict the relative impact of possible determinants of synesthetic colors, such as ordinality, phonology, and visual shapes, and draw a comprehensive picture of grapheme-color association process in synesthesia. The purpose of this study is to test the validity of this model by examining its predictions of the relative impacts of several factors on synesthetic color selection. To this end, we predicted the interrelationships of several possible determinants of synesthetic colors in Japanese Hiragana characters; in addition these predictions were tested in a behavioral experiment using synesthetes whose native language is Japanese. We chose Hiragana characters because, according to model predictions, there are more grapheme properties that can have significant (observable) impact on grapheme color associations than in the case of English alphabet letters. This will enable a more efficient test of the model. We chose to evaluate Hiragana characters rather than Katakana characters (which are also phonetic characters in the Japanese language with similar properties as Hiragana characters) because Hiragana characters are the graphemes that most native Japanese speakers learn first (analogously, letters of the English alphabet are graphemes learned first by most native English speakers). Katakana characters are usually learned after Hiragana characters (see Asano and Yokosawa, 2011, 2012).

The model leads to the following central hypothesis: Synesthetic colors for Hiragana characters are greatly influenced by ordinality and sounds (phonemes), whereas synesthetic colors for the English alphabet are greatly influenced by ordinality, but not by sounds (phonemes). In both cases the model predicts that synesthesia is weakly affected by visual shapes.

The rationale behind this hypothesis holds that among the three feature domains of interest (sound, ordinality, shapes), sounds should have the weakest impact on synesthetic colors with the English alphabet because one grapheme is usually arbitrarily associated with several phoneme representations. In addition to synesthetic colors for Hiragana characters, we also examined those for English alphabet letters in Japanese synesthetes for a further test of the model. Although the English alphabet is not a writing system for their mother tongue (i.e., Japanese), most Japanese people learn it at around the ages of 9–10 years in elementary school and have no difficulty in reading and writing English alphabet letters. The English alphabet is first introduced to Japanese children as a sequence (i.e., the ABC sequence) and then they learn to represent sounds of Japanese words using the English alphabet (referred to as Roma-ji). At that time, children notice that the pronunciations of letter names and that of letters are not the same in the English alphabet as well as that some letters (e.g., L, Q, X) are not used to represent Japanese sounds because there are no corresponding sounds in the Japanese language. All of these considerations figured into our main prediction that synesthetic colors for the English alphabet will be greatly influenced by ordinality, and less affected by visual shapes, and least affected by sounds even in Japanese synesthetes.

To test model predictions, we used multiple regression analyses to assess the relative impact of ordinality, sound, visual shape, and grapheme familiarity on synesthetic color choice for Hiragana characters and English alphabet letters. We included grapheme familiarity in addition to the other three predictors, although familiarity is not referred in the model (cf. Figure 1). This is because there have been a few reports of some influence of grapheme frequency on synesthetic colors (e.g., Beeli et al., 2007; Watson et al., 2012). Grapheme familiarity is closely related to grapheme frequency. These two factors differ in that familiarity refers to subjective familiarity ratings of graphemes whereas grapheme frequency refers to grapheme norms in a defined corpus of publications, such as newspapers. We used subjective familiarity instead of frequency because it should more validly reflect processing efficiencies of the characters/letters in the brain of young children, who have not yet been exposed to the published literature.

We employed the idea of second-order similarity mappings in grapheme-color synesthesia proposed by Watson et al. (2012). Second-order mappings refer to correlations between differences in values in one domain of grapheme features and differences in synesthetic colors of graphemes (e.g., more visually similar letters tend to be associated with more similar colors). This is contrasted to the idea of first-order mappings, which are correlations between absolute (non-relational) properties of a letter and dimensions of synesthetic color (e.g., the letter “y” tends to be yellow, because “y” is for the word “yellow”). Second-order mapping analysis is suitable for examining the influence of the four grapheme properties on synesthetic colors, ordinality, phonology, visual shapes, and grapheme familiarity, because all the four of these properties can be described relationally (i.e., more similar/different). Examining relations involving differences between graphemes and their assigned colors allowed us to directly compare and contrast multiple influences on synesthetic grapheme-color associations. Whereas Watson and his colleagues examined the independent effects of grapheme properties (e.g., letter shape, frequency, and ordinality) on grapheme-color associations in synesthesia, in this study, we focused on the *relative* impact of these properties. The aim was to test our hypothesis that synesthetic colors are associated with graphemes during grapheme acquisition, and that the color for the representation of the feature which makes a *relatively large* contribution in discriminating the grapheme from others is the color most likely to be ultimately associated with the grapheme. We believe we can provide a comprehensive picture of grapheme-color association in synesthesia by focusing on the relative impact of grapheme properties and considering the ways in which graphemes are introduced to children during development.

## Materials and methods

### Participants

Seventeen Japanese grapheme-color synesthetes (16 female, one male, mean age = 25.0 years, *SD* = 7.2 years), whose first language is Japanese, participated in this study. All reported experiencing synesthetic colors when viewing Hiragana characters and English alphabet letters, as well when viewing Katakana and Kanji characters and Arabic numerals (i.e., digits). All reported that auditory input of linguistic sounds does not elicit synesthetic experiences (unless converted to graphemes). Informed consent was obtained from all participants of this study after the nature and possible consequences of the studies were explained. The rights of the participants were protected. All the experimental procedures had been approved by the Ethical Committee of Department of Psychology, Graduate School of Humanities and Sociology, The University of Tokyo in Japan.

To establish the genuineness of participating synesthetes, we also recruited six female non-synesthetic control participants; mean age was 22.3 years (*SD* = 2.6 years). All were undergraduate or graduate students from universities in Japan and were native speakers of Japanese.

### Apparatus

Characters and a color palette were displayed on a monitor (Mitsubishi Diamondtron M2 RDF223G). One hundred and thirty-eight HTML named colors (colors supported by major web browsers) were in the palette (Asano and Yokosawa, 2011, 2012). We chose this color set because a palette should consist of common and mutually distinguishable colors. Each of the 138 colors has its own name, therefore we regard the palette as a fine category set of colors which is used conventionally and universally. The palette incorporated a variety of colors while reducing the requisite effort in selecting colors (compared to the use of an extensive color palette as in Eagleman et al. (2007), which has 256 × 256 × 256 colors). Note, however, that use of the 138-color palette may reduce the sensitivity of the experimental results to the real effects of various factors on color-grapheme synesthesia. Color coordinates in the CIE L*a*b* systems, used in analyses of the results, were converted from the CIE xyY coordinates of presented colors; all were measured using Topcon BM-7 luminance colorimeter. We adopted the CIE L*a*b* system because it was designed in such a way that mathematical differences in all color ranges correspond to perceived color differences (Kaatsch et al., 1993). We further converted the color coordinates in the CIE L*a*b* system to coordinates in the CIE L*C*h system in order to obtain hue, saturation, and luminance values.

### Stimuli

Hiragana characters: Among the entire set of 71 Hiragana characters, all 46 basic characters were used as stimuli^2^. Five of these were symbols for single vowels (/a/, /i/, /u/, /e/, and /o/). Of the remaining characters, 40 were symbols for syllables that is, combinations of a consonant (/k/, /s/, /t/, /n/, /h/, /m/, /y/, /r/, /w/) and one of the five vowels (no characters for syllables /yi/, /ye/, /wi/, /wu/, /we/ exist in the Japanese language). The remaining character represents the sound of /n/, which has no vowel.

English alphabet letters: The entire set of 26 English uppercase alphabet letters were employed as stimuli.

### Procedure

While seated in a darkened room, each synesthete used a computer mouse to select a palette color corresponding to one of the stimulus characters/letters. Synesthetes were told to select a color closest to their synesthetic colors. They were also told to select the color black if a presented character elicited a sensation of the color black or no synesthetic color. The Hiragana characters and English alphabet letters were presented (for color selection) in a randomized order in respectively, separate blocks in a session. No characters/letters were repeated within a session.

Two temporally separated experimental sessions were presented to participants in order to test the consistency of their color selections over time (Eagleman et al., 2007). The stimuli were the same, but the order of character/letter appearance differed randomly between the two sessions. Sessions were separated by intervals of at least 3 weeks (mean interval = 10.1 weeks, *SD* = 11.2 weeks). The averages of color distances (Euclidean distance in the CIE L*a*b*) for colors selected for a given character/letter in the first and second session, calculated and averaged separately for the 46 Hiragana characters and the 26 English alphabet letters, were very small; 19.7 (*SE* = 1.4, range = 10.3–31.3) for Hiragana characters, and 16.3 (*SE* = 1.2, range = 6.8–25.3) for English alphabet letters. These values are strikingly small given that the average color distance for random color pairs from the 138 colors in the palette (i.e., chance level) was 67.9 (*SD* = 31.8) and analogous values obtained from six non-synesthetic controls, who engaged in the same task as synesthetic participants with 2 weeks intervals between the two sessions, were 62.4 (*SE* = 7.2, range = 33.3–86.2) for Hiragana characters and 65.8 (*SE* = 7.7, range = 29.2–86.1) for English alphabets. An unequal variance *t*-test (Welch's method, two-tailed) revealed that the mean color distances across sessions for the synesthete group were significantly smaller than those for the control group, *t*_(5)_ = 5.34 for Hiragana characters and *t*_(5)_ = 5.83 for English alphabet letters, both *p*s < 0.01, Bonferroni corrected. These results clearly demonstrate that participants engaged in this study were genuine synesthetes. Data from the first session was used for the following analyses.

### Data preparation

Using the method proposed by Watson et al. (2012), we explored second-order similarity mappings. In addition to color distance (Euclidean distance in CIE L*a*b* color space), we computed luminance distance, saturation distance (difference of C* values in the CIE L*C*h color coordinate) and hue distance (difference of *h*-values in the CIE L*C*h color coordinate) between two graphemes separately as color difference measures. Four dependent measures of grapheme difference/similarity were compared with each of the synesthetic color difference measures: ordinality difference, phonological similarity, visual shape similarity, and grapheme familiarity difference. Ordinality difference refers to a difference between the positions in the Hiragana/alphabet sequence of two characters/letters, divided by their sum. Phonological similarity refers to the number of shared phonemes (consonants and vowels) in names of two characters/letters. Graphemes names are exactly the same as the pronunciation in the case of Hiragana, but this is not in the case for letters in the English alphabet. Visual similarity refers to subjective comparison ratings of the similarities of two characters/letters in Hiragana (Kawakami and Tsuji, 2012) and the English alphabet (Boles and Clifford, 1989), both rated on a five-point scale from 1 (low similarity) to 5 (high similarity). Character/letter familiarity differences refer to differences between the subjective familiarity ratings of two Hiragana characters/English alphabet letters, both rated by adult native Japanese speakers in a Japanese language environment on a seven-point scale from 1 (low familiarity) to 7 (high familiarity) (Amano and Kondo, 1999).

There were 1035 and 325 possible Hiragana character and English alphabet letter pairs, respectively (not including doubles of the same character/letter). We computed separate values for color distance, luminance distance, saturation distance, and hue distance for each of these pairs. All subsequent analyses were performed after binning the 1035 Hiragana character pairs into 69 bins that each included 15 character pairs. For 325 English alphabet letter pairs 65 bins were used, each included 5 letter pairs^3^ (see Watson et al., 2012, for the original method). Bins were determined by the mean color distance of each character/letter pair across all 17 synesthetes, such that the first bin of the Hiragana character pairs and English alphabet pairs, respectively, contained pairs whose two characters/letters were, on average, most similar in color.

## Results

### Hiragana characters

Correlations among variables for Hiragana characters are shown in Table 1. To test for multicollinearity among independent variables, we examined the variance inflation factor (VIF). The VIF values were all below 1.5, indicating multi-collinearity was not an issue. See Figure S1 in Supplementary Materials for a visualization of distance matrices of each color/grapheme property measure for Hiragana characters.

**Table 1:** **Correlations among the variables for Hiragana characters (*N* = 69)**.

	**Color dist.**	**Lum. dist.**	**Sat. dist.**	**Hue dist.**	**Ord. diff.**	**Phono. sim.**	**Shape sim.**
Color distance							
Luminance distance	0.79^**^						
Saturation distance	0.93^**^	0.80^**^					
Hue distance	0.96^**^	0.70^**^	0.82^**^				
Ordinality difference	0.74^**^	0.58^**^	0.67^**^	0.71^**^			
Phonological similarity	−0.71^**^	−0.58^**^	−0.67^**^	−0.74^**^	−0.46^**^		
Shape similarity	−0.58^**^	−0.54^**^	−0.61^**^	−0.54^**^	−0.33^**^	0.53^**^	
Familiarity difference	0.61^**^	0.34^**^	0.59^**^	0.55^**^	0.54^**^	−0.38^**^	−0.24

** *p < 0.01*.

As shown in Table 2, a multiple regression model using the four independent variables (ordinality difference, phonological similarity, shape similarity, and familiarity difference) as predictor variables for color distance in synesthetic colors for Hiragana characters [*R*^2^ = 0.79, *F*_(4, 64)_ = 59.66, *p* < 0.01]. This analysis also revealed that ordinality difference was the strongest predictor of synesthetic color distance (Beta = 0.394, *p* < 0.01), followed by phonological similarity (Beta = −0.333, *p* < 0.01), shape similarity (Beta = −0.225, *p* < 0.01), and familiarity difference (Beta = 0.213, *p* < 0.01). Overall, this analysis supports our hypothesis, which predicted a large impact of ordinality and phonology, and a relatively small impact of visual shape on synesthetic colors for Hiragana characters.

**Table 2:** **Results of multiple regression analyses for Hiragana character pairs**.

**Variables**	**Unstandardized coefficient**		**Standardized coefficient**	***t*-value**	***p*-value**
	***B***	***SE***	**β**		
**COLOR DISTANCE [*R*^2^ = 0.79, *F*_(4, 64)_ = 59.66, *p* < 0.01]**					
Ordinality difference	45.972	8.492	0.394	5.41	<0.01
Phonological similarity	−24.541	5.415	−0.333	−4.53	<0.01
Shape similarity	−19.430	5.899	−0.225	−3.29	<0.01
Familiarity difference	48.743	15.902	0.213	3.07	<0.01
**LUMINANCE DISTANCE [*R*^2^ = 0.51, *F*_(4, 64)_ = 16.90, *p* < 0.01]**					
Ordinality difference	14.599	4.176	0.386	3.50	<0.01
Phonological similarity	−6.400	2.663	−0.268	−2.40	<0.05
Shape similarity	−7.706	2.901	−0.276	−2.66	<0.01
Familiarity difference	−2.663	7.820	−0.036	−0.34	0.73
**SATURATION DISTANCE [*R*^2^ = 0.73, *F*_(4, 64)_ = 42.27, *p* < 0.01]**					
Ordinality difference	13.420	3.563	0.312	3.76	<0.01
Phonological similarity	−7.283	2.272	−0.269	−3.20	<0.01
Shape similarity	−9.786	2.475	−0.308	−3.95	<0.01
Familiarity difference	20.907	6.671	−0.248	3.13	<0.01
**HUE DISTANCE [*R*^2^ = 0.74, *F*_(4, 64)_ = 46.18, *p* < 0.01]**					
Ordinality difference	46.218	9.641	0.385	4.79	<0.01
Phonological similarity	−32.336	6.147	−0.427	−5.26	<0.01
Shape similarity	−13.221	6.697	−0.149	−1.97	0.05
Familiarity difference	33.233	18.053	0.141	1.84	0.07

SE, standard error.

We also conducted multiple regression analyses to predict luminance, saturation, and hue distances separately using the same four independent variables (Table 2). A regression model using the four independent variables to predict luminance distance in synesthetic colors for Hiragana characters [*R*^2^ = 0.51, *F*_(4, 64)_ = 16.90, *p* < 0.01] also revealed that ordinality difference was the strongest predictor (Beta = 0.386, *p* < 0.01), followed by shape similarity (Beta = −0.276, *p* < 0.01) and phonological similarity (Beta = −0.268, *p* < 0.05). Luminance distance was not significantly predicted by familiarity difference (Beta = −0.036, *p* = 0.73). A similar trend was observed in the multiple regression analysis for saturation distance [*R*^2^ = 0.73, *F*_(4, 64)_ = 42.27, *p* < 0.01]; ordinality difference was the strongest predictor (Beta = 0.312, *p* < 0.01), followed by shape similarity (Beta = −0.308, *p* < 0.01) and phonological similarity (Beta = −0.269, *p* < 0.01). Familiarity difference also significantly predicted saturation distance (Beta = −0.247, *p* < 0.01). A regression model to predict hue distance [*R*^2^ = 0.74, *F*_(4, 64)_ = 46.18, *p* < 0.01] showed hue distance was significantly predicted only by phonological similarity (Beta = −0.427, *p* < 0.01) and ordinality difference (Beta = 0.385, *p* < 0.01). In addition to this, shape similarity and familiarity difference each levied only a marginally significant effect on predicted hue distance (Beta = −0.149, *p* = 0.05; Beta = 0.141, *p* = 0.07, respectively).

### English alphabet letters

Correlations among variables for English alphabet letters are shown in Table 3. The VIF values were all below 1.1, indicating multicollinearity was not an issue. See Figure S2 in Supplementary Materials for visualized distance matrices of each color/grapheme property measures for English alphabetical letters.

**Table 3:** **Correlations among the variables for English alphabet letter pairs (*N* = 65)**.

	**Color dist.**	**Lum. dist.**	**Sat. dist.**	**Hue dist.**	**Ord. diff.**	**Phono. sim.**	**Shape sim.**
Color distance							
Luminance distance	0.58^**^						
Saturation distance	0.57^**^	0.52^**^					
Hue distance	0.88^**^	0.42^**^	0.44^**^				
Ordinality difference	0.43^**^	0.19	0.27^*^	0.28^*^			
Phonological similarity	0.15	0.21	−0.04	0.08	−0.05		
Shape similarity	−0.32^*^	−0.25^*^	−0.14	−0.32^*^	−0.22	−0.03	
Familiarity difference	0.15	0.13	0.18	0.05	0.04	0.01	−0.30^*^

* p < 0.05,

** p < 0.01.

Table 4 shows the results of a multiple regression model using the four independent variables as predictors for color distance in synesthetic colors for English alphabets [*R*^2^ = 0.27, *F*_(4, 60)_ = 5.50, *p* < 0.01]. It reveals that ordinality difference was the only statistically significant predictor (Beta = 0.393, *p* < 0.01); shape similarity had a marginally significant effect on predicted hue distance (Beta = −0.201, *p* = 0.10).

**Table 4:** **Results of multiple regression analyses for English alphabet letter pairs**.

**Variables**	**Unstandardized coefficient**		**Standardized coefficient**	***t*-value**	***p*-value**
	***B***	***SE***	β		
**COLOR DISTANCE [*R*^2^ = 0.27, *F*_(4, 60)_ = 5.50, *p* < 0.01]**					
Ordinality difference	33.614	9.707	0.393	3.46	<0.01
Phonological similarity	7.044	4.947	0.158	1.42	0.16
Shape similarity	−11.541	6.831	−0.201	−1.69	0.10
Familiarity difference	11.193	17.271	0.075	0.65	0.52
**LUMINANCE DISTANCE [*R*^2^ = 0.13, *F*_(4, 60)_ = 2.24, *p* = 0.08]**					
−			−	−	−
**SATURATION DISTANCE [*R*^2^ = 0.11, *F*_(4, 60)_ = 1.76, *p* = 0.15]**					
−			−	−	−
**HUE DISTANCE [*R*^2^ = 0.16, *F*_(4, 60)_ = 2.76, *p* < 0.05]**					
Ordinality difference	23.310	12.793	0.222	1.82	0.07
Phonological similarity	4.380	6.520	0.080	0.67	0.50
Shape similarity	−19.753	9.002	−0.280	−2.19	<0.05
Familiarity difference	−7.870	22.762	−0.043	−0.35	0.73

SE, standard error.

We also conducted multiple regression analyses to predict luminance, saturation, and hue distances of synesthetic colors for English alphabet letters, respectively, with the four independent variables (Table 4); however, multiple regression models to predict luminance distance and saturation distance were not statistically significant [*R*^2^ = 0.13, *F*_(4, 60)_ = 2.24, *p* = 0.08; *R*^2^ = 0.11, *F*_(4, 60)_ = 1.76, *p* = 0.15, respectively]. The regression model for hue distance was significant [*R*^2^ = 0.16, *F*_(4, 60)_ = 2.76, *p* < 0.05], and it was shown that shape similarity significantly predicted hue distance (Beta = −0.280, *p* < 0.05); the ordinality difference had only a marginally significant effect on predicted hue distance (Beta = 0.217, *p* = 0.08).

## Discussion

### Summary of the findings

The results for Hiragana characters, in general, supported our predictions. As predicted, a multiple regression analysis revealed that ordinality difference and phonological similarity were strong predictors (Beta = 0.394, −0.333, respectively) of synesthetic color distances for Hiragana characters. The impact of visual shape similarity, although also statistically significant, was relatively small (Beta = 0.225). The impact of both phonology and ordinality was manifest in the dimension of hue of synesthetic colors, whereas the impact of visual shape and grapheme familiarity on the hue dimension was relatively small (statistically marginally significant). Ordinality difference was not only the strongest predictor of color distance; it was also dominant in predicting luminance, and saturation. The impact of visual shape similarity was comparable to that of phonological similarity in the luminance dimension and greater than that of phonological similarity in the saturation dimension, both of which were slightly different from our prediction. Grapheme familiarity difference affected color distance and saturation of synesthetic colors significantly. Thus, we observed significant influence of visual shape on synesthetic colors. This finding differs from that reported by Asano and Yokosawa (2011), who found no effect of visual shape on synesthetic colors for Hiragana characters. This inconsistency can be explained as follows. Asano and Yokosawa (2011) concluded that synesthetic color selection for Hiragana characters did not depend on visual shape based on the fact that synesthetic colors for two characters sharing the same sounds, namely a Hiragana character and its Katakana counterpart, were found to be remarkably consistent regardless of the differences in their shapes. That is, Asano and Yokosawa compared visual shapes of two characters from respectively different sets of graphemes. The presence of visual shape effects in the present results suggests that it is *relative* shape similarity within a set of graphemes that affects synesthetic color similarity rather than the *absolute* similarity/difference of visual images. In other words, relations between graphemes are measured independently across different sets of graphemes.

The results for English alphabet letters were also generally in accordance with the predictions of the model proposed in this study. We predicted a strong influence of ordinality for these stimuli and a weaker influence of visual shape. Finally, phonology was predicted to have the weakest influence on synesthetic colors for English alphabet letters. The results showed that statistically the ordinality difference served as a significant predictor of color distance and that there was also a marginally significant impact of shape similarity; however, there was no significant impact of phonology. Moreover, visual shape similarity proved to be a statistically significant predictor of hue and there was also a marginally significant impact of ordinality difference; again there was no significant impact of phonology. However, the explanatory powers of the models for English alphabet letters were generally small (at most *R*^2^ = 0.27, in the case of color distance) compared to those for Hiragana characters (*R*^2^ = 0.51~0.79).

### Congruence of categorical vs. continuous properties between graphemes and colors

Watson et al. (2012) conjectured that a congruency factor can explain certain selective effects observed in second-order relations among grapheme-color pairs. They examined various associations between synesthetic colors and English alphabet letters (in synesthetes who were probably English speakers, although their native language was not specified in Watson et al., 2012). Of most interest, they proposed that grapheme properties that are processed categorically (e.g., letter shape) are mapped to a congruent, i.e., categorically perceived, color dimension (i.e., hue). Similarly, they proposed that grapheme properties that are processed continuously (e.g., frequency) are mapped to color dimensions that are perceived as continuums (i.e., luminance and saturation). In their study, shape difference (calculated based on 11-dimensional distinctive letter features proposed by Gibson, 1969) was found to correlate significantly with color and hue distance, and frequency difference significantly correlated with luminance distance; in addition, the ordinality difference significantly correlated with color and hue distance. They explained the correlation between ordinality difference and hue by assuming that ordinality may be processed categorically, although it varies continuously, because it is used as a rough index of the order of learning of individual letters.

Although Watson et al. (2012) examined synesthesia associated with English letters, their interpretations offer some generality which may facilitate understanding findings with Hiragana characters in the present study. In terms of congruence of categorical vs. continuous properties, we observed a significant influence of grapheme familiarity, which varies continuously, on color distance and saturation distance, both of which also (at least in some part) vary continuously, among Hiragana characters. Phonology, which is a strictly categorical entity, was the strongest predictor of hue distance, and it is often perceived categorically. However, ordinality strongly affected all the dimensions of synesthetic colors, both categorical (i.e., hue, and color distance in some part) and non-categorical (i.e., luminance, saturation, and color distance in some part). The latter outcome may suggest that, for some reasons, both categorical and continuous aspects of ordinality are reflected to synesthetic colors for Hiragana characters. Part of the reason may be the fact that there are more Hiragana characters (46 basic characters, 71 characters in total) than letters in the English alphabet (26 characters). The number of Hiragana characters may be sufficiently large such that it renders it difficult to process all the characters categorically (e.g., to use ordinality as a rough index of the order of learning of each grapheme); thus continuous aspects of ordinality may be relatively emphasized.

### Which aspects of visual shape matter?

The results of Watson et al. (2012) and those of the present study differ in a few respects. In particular visual shape influenced hue of synesthetic colors for English alphabet letters in the study of Watson et al. (suggesting that visual shapes are processed categorically). By contrast, in our study visual shape affected luminance, saturation, and color distance among synesthetic colors for Hiragana characters (suggesting that visual shapes are processed continuously), and it also had a marginally significant effect on hue distance (suggesting that visual shapes are processed categorically). This may be due to the differences between the two sets of graphemes. Because the number of graphemes is greater in the Hiragana syllabary than it is in the English alphabet, and because Hiragana characters are generally visually more complex than the English alphabet, some aspects of visual shapes of Hiragana characters may be processed more quantitatively as compared with processing in the English alphabet. For example, not only the presence/absence of basic visual features (i.e., categorical, qualitative properties) but also properties such as relative length of components and number of strokes (i.e., quantitative properties) may have strong impact on Hiragana character discrimination.

The results for English alphabet letters in the present study are similar to those reported by Watson et al. (2012) using English speakers. For example, visual shape in both studies affected hues. However, we must acknowledge that the results differ in some respects. As visual shape similarity/difference measure(s), we used subject letter similarity ratings from Boles and Clifford (1989) in the present study, whereas Watson et al. (2012) used several different measures including both the letter similarity ratings (from Boles and Clifford, 1989) and the shape difference calculated based on 11-dimensional distinctive letter features proposed by Gibson (1969). Thus, the similarity ratings from Boles and Clifford (1989) were used in both studies. However, for some reason, this predictor significantly affected hues in the present study but not in the Watson et al. (2012) study. Instead, in Watson et al. (2012), as noted above, the shape difference measure, calculated using the basic letter shape features from Gibson (1969) significantly affected hues.

To assess the impact of the shape difference measure (based on Gibson, 1969) on synesthetic colors for English alphabet letters reported by Japanese synesthetes in the present study, we conducted additional multiple regression analyses. We sought to predict color, luminance, saturation, and hue distance in synesthetic colors for English alphabet letters found in present study using ordinality difference, phonological similarity, familiarity, and the visual shape difference (calculated based on Gibson, 1969, instead of subjective shape similarity provided by Boles and Clifford, 1989) as predictors^4^. The overall results were similar to those of the multiple regression analyses in the present study (see Results) in which the Boles and Clifford's measure was used as the visual shape similarity measure: multiple regression models to predict luminance distance and saturation distance were not statistically significant [*R*^2^ = 0.13, *F*_(4, 60)_ = 2.18, *p* = 0.08; *R*^2^ = 0.12, *F*_(4, 60)_ = 1.98, *p* = 0.11, respectively]. The regression model for hue distance and color distance were significant [*R*^2^ = 0.28, *F*_(4, 60)_ = 5.97, *p* < 0.01; *R*^2^ = 0.32, *F*_(4, 60)_ = 7.00, *p* < 0.01, respectively], and it was shown that hue distance was significantly predicted by the shape difference (Beta = 0.462, *p* < 0.01), and color distance was significantly predicted by ordinality difference and the shape difference (Beta = 0.394, 0.303, respectively, both *p*s < 0.01). Note that the explanatory powers of the models are greater when the Gibson's measure was used than when the Boles and Clifford's measure was used (hue distance: Boles and Cliffor's measure *R*^2^ = 0.16, Gibson's measure *R*^2^ = 0.28; color distance: Boles and Cliffor's measure *R*^2^ = 0.27, Gibson's measure *R*^2^ = 0.32), which may be in accordance with Watson et al. (2012) in which significant effects of the Gibson's measure but not Boles and Clifford's measure were observed. Since Gibson (1969) considered neurophysiological and developmental observations in selecting the distinctive letter features while the Boles and Clifford's measure, which consists of ratings on subjective letter similarity by adults and may reflect various information associated to the letters such as knowledge, it may be these types of neurophysiological processing of basic visual properties in childhood that affect synesthetic color associations for English alphabet letters. This is in accordance with the model proposed in this study.

### Synesthetic colors as “discriminating markers” of graphemes during learning graphemes

The model we have proposed explains the synesthetic color for Hiragana characters quite well (79 and 74% of the variance was predicted by the four variables in the case of color distance and hue distance, respectively). However, its explanatory power declined for the English alphabet letters in this study (only 27 and 16% of the variance was predicted in the case of color distance and hue distance, respectively). Since the number of grapheme pairs included in a bin in the second-order mapping analyses differed between Hiragana characters and English alphabet letters (15 and 5 pairs, respectively. See footnote 3 for more information), it may be difficult to make direct comparisons of explanatory powers of the models between Hiragana characters and English alphabet letters. Even taking this into account, the model proposed in this study still appears to explain the synesthetic color for Hiragana characters better than it explains synesthetic color for English alphabet letters; the results showed that the phonological similarity had a strong effect in Hiragana characters but not in English alphabets, which means that the model revealed more predictors in the case of Hiragana characters.

One possible reason for the low explanatory power for English alphabet letters in this study is that the English alphabet is not the writing system of the native language of our participants and they are not very familiar with its letters. According to a corpus of a major newspaper, the mean frequency of 26 English alphabet letters is roughly 1/42 of the mean frequency of 46 basic Hiragana characters (Amano and Kondo, 2000). The lack of influence of letter familiarity on synesthetic colors for English alphabet letters in this study supports this idea (i.e., letter familiarity was too low in general to affect synesthetic color choices). Another possible reason for this result is that the synesthetes who participated in this study had already acquired several sets of graphemes (e.g., Hiragana, Katakana, and some Kanji characters) prior to acquiring letters in the English alphabet. As previous studies have shown, synesthetic colors for graphemes acquired early in life can transfer to those for graphemes acquired later in life (Mills et al., 2002, 2009; Witthoft and Winawer, 2006; Asano and Yokosawa, 2012). Since the present model omits such transfer effects of synesthetic colors from other sets of graphemes, any already-acquired mappings between Hiragana characters and synesthetic colors might have disrupted the expected patterns of results in English grapheme-color associations. However, we do not think these are the only reasons for the low explanatory power of the model for the English alphabet letters in this study. Watson et al. (2012) conducted multiple regression analyses to predict hue distance with 11 predictors including shape similarity/difference (including Gibson's and Boles and Clifford's measures), letter frequency difference, ordinality difference, phonological (letter name) similarity. He found that even with native English speakers and even using as many as 11 predictors, the 11 predictors explained at most about 32% of the variance (details of this analysis were not supplied). Given that the participants in the study of Watson et al. (2012) were highly familiar with English alphabet letters, the reduced explanatory power of these regression models cannot be attributed to letter familiarity.

We propose a different explanation for the generally low explanatory powers of the second-order measures with the English alphabet. It hypothesizes that a synesthetic color highlights the most discriminating feature of each grapheme, which people (both synesthetic and non-synesthetic) rely on when they learn graphemes. In turn, this “marking” potential facilitates people's discrimination of a grapheme which then leads to their speedier learning of that grapheme. In doing so, we assume that not all properties of a given grapheme afford this “marking” potential nor do these markers apply to all graphemes. For example, ordinal numbers may provide effective discriminating cues for certain graphemes in a sequence i.e., from the first to the fifth, at most. However, it should be difficult to associate the larger ordinal numbers with successive graphemes. Other than the cases of the graphemes above (i.e., from the first to the fifth graphemes), information provided by the domain of ordinality may be rough, like either “early” or “later” in the sequence. This means that ordinality can afford effective “marking” potential only in some graphemes (e.g., the first to the fifth graphemes in a grapheme sequence, and graphemes that appear distinctively early or late in a sequence); furthermore, it should be difficult to distinguish all graphemes with assistance of only ordinality information. Similarly, it is likely that each of phonology, visual shape, and familiarity can afford effective “marking” potential only in some (different) graphemes. This reasoning leads us to hypothesize that multiple basic grapheme properties need to be utilized simultaneously to attach a “marker” to each of all graphemes.

We presume that certain basic grapheme properties such as ordinality, phonology, visual shape are preferentially used (when available) as “markers” in grapheme discrimination. The rationale of this is that they should be highly stable in the input to children because such properties are socially shared. If some of the basic grapheme properties are not useful in discriminating and learning graphemes (as in the case of phonology in discriminating English alphabet letters), children need to find other sources of information available for grapheme discrimination. Such additional sources might involve one's memory for properties associated with graphemes (e.g., colors of refrigerator magnets), word meanings (e.g., “ ‘B’ is for ‘banana’ so the color of ‘b’ is yellow”), and so on. The additional sources will usually differ across individuals because they depend on personal experiences.

Note that the basic properties are relational, while the additional sources may usually have non-relational properties. We argue that this is the reason for the generally low explanatory powers of the second-order measures with the English alphabet in the present study and in Watson et al. (2012). Fewer relational basic grapheme properties are available in English alphabet letter discrimination due to the fact that phonology does not provide effective discrimination cues. This suggests that synesthetic colors for English alphabet letters may depend more on non-relational properties such as memories associated with certain graphemes (e.g., Witthoft and Winawer, 2006, 2013) and word meanings (e.g., Rich et al., 2005; Simner, 2007) than do these colors for Japanese Hiragana characters. Since the analyses in this study and in Watson et al. (2012) were designed to examine second-order relationships between relational grapheme properties and synesthetic colors, we conjecture that the regression models in these studies had only limited powers for explaining grapheme-color associations in synesthesia.

## Concluding remarks

We have proposed a new model that provides a comprehensive explanation of synesthetic grapheme-color association determination processes, including the differences across writing systems. It is based upon previous studies on developmental perspective of grapheme-color synesthesia (Rich et al., 2005; Asano and Yokosawa, 2012; Watson et al., 2012). Results of an accompanying experiment on synesthetic colors for Hiragana characters and English alphabet letters were generally consistent with the model. Together model and results suggest that the ways in which graphemes are introduced to children during development and are processed in the brain are critical. Indeed, these factors are more important in determining grapheme-color associations than grapheme properties *per se*. This challenges a view that synesthesia requires unusual hard-wired cross-associations between certain brain areas (e.g., Brang et al., 2010, 2011). The present model is partly consistent that of Brang et al. (2011) in assuming that some aspects of synesthetic colors are associated with basic visual components of letters in some ways. However, it differs in supporting the view that grapheme-color synesthesia builds on normal cognitive (including language) processing mechanisms (Simner et al., 2005; Simner, 2007; Asano and Yokosawa, 2011, 2012; Watson et al., 2012)).

The model of the present study assumes that linkages between color representations and representations of features in some domains (e.g., ordinality in conventional sequences, sounds, and visual shapes) are formed in the brain before grapheme acquisition. We believe this is possible based on the observations reported in several previous studies (e.g., Rich et al., 2005; Wagner and Dobkins, 2011; Asano and Yokosawa, 2012; see Introduction), however, direct evidence for this must await further studies.

According to the model proposed in this study, synesthetic colors function as “discriminating markers” of graphemes during learning graphemes, which are arbitrary symbols that may not be easily acquired by children. Certain questions are left unanswered: why colors are used as “markers”? How this is achieved in the brain? If the “discrimination markers” view holds true, why are colors also associated with other cognitive entities that people do not have to “acquire” (meaning that no special training is required to process them), such as music sounds and tastes, in other types of synesthesia, such as colored hearing and gustatory-color synesthesia? Are these types of synesthesia qualitatively different from grapheme-color synesthesia? Future studies are required.

## Author contributions

Michiko Asano and Kazuhiko Yokosawa designed the study and collected data. Michiko Asano analyzed the data and wrote the paper. Kazuhiko Yokosawa reviewed and refined the paper. Both authors discussed the results and commented on the manuscript.

### Conflict of interest statement

The authors declare that the research was conducted in the absence of any commercial or financial relationships that could be construed as a potential conflict of interest.

## Footnotes

^1^More precisely, one Hiragana character is linked to either one phoneme or one pair of phonemes, because Hiragana script is a syllabary representing vowels or syllables (i.e., combinations of a consonant and a vowel). Nonetheless, we call this “one-to-one relationship between graphemes and phonemes” in this article for convenience. The point here is that grapheme-phoneme correspondence is transparent in Hiragana characters.

^2^The remaining 25 non-basic characters are so-called “dakuon” and “handakuon” characters in the Japanese language. These characters were not used as stimuli in this study because their positions in the Hiragana sequence are not well established and subject to change depending on circumstances (e.g., the index in a dictionary, word sorting functions on a computer). The basic and non-basic Hiragana characters are usually appear in separate tables in educational materials.

^3^The number of pairs included in a bin differed between Hiragana characters (15 pairs) and English alphabet letters (5 pairs). This was to match the numbers of items used in each multiple regression analysis between the two scripts as closely as possible. The overall results for Hiragana characters were almost the same between when 5 pairs were included in a bin (i.e., 207 bins were used for multiple regression analyses. See Tables S2, S3 in Supplementary Materials for detailed results) and when 15 pairs were included in a bin (see Table 2 in the text).

^4^Unfortunately, visual shape difference measures for Hiragana characters which are analogous to the Gibson's measure do not exist. Hiragana characters are visually too complicated to classify based on the existence/absence of simple basic visual features. Therefore we could not conduct analogous analyses for Hiragana characters in this study.
