# Supplementary material for: Grapheme learning and grapheme-color synesthesia: toward a comprehensive model of grapheme-color association
Source: Front Hum Neurosci. 2013 Nov 11;7:757. doi: 10.3389/fnhum.2013.00757 (PMC3822291; doi:10.3389/fnhum.2013.00757)
Supplement: Supplementary file 2 [file Presentation2.PDF]

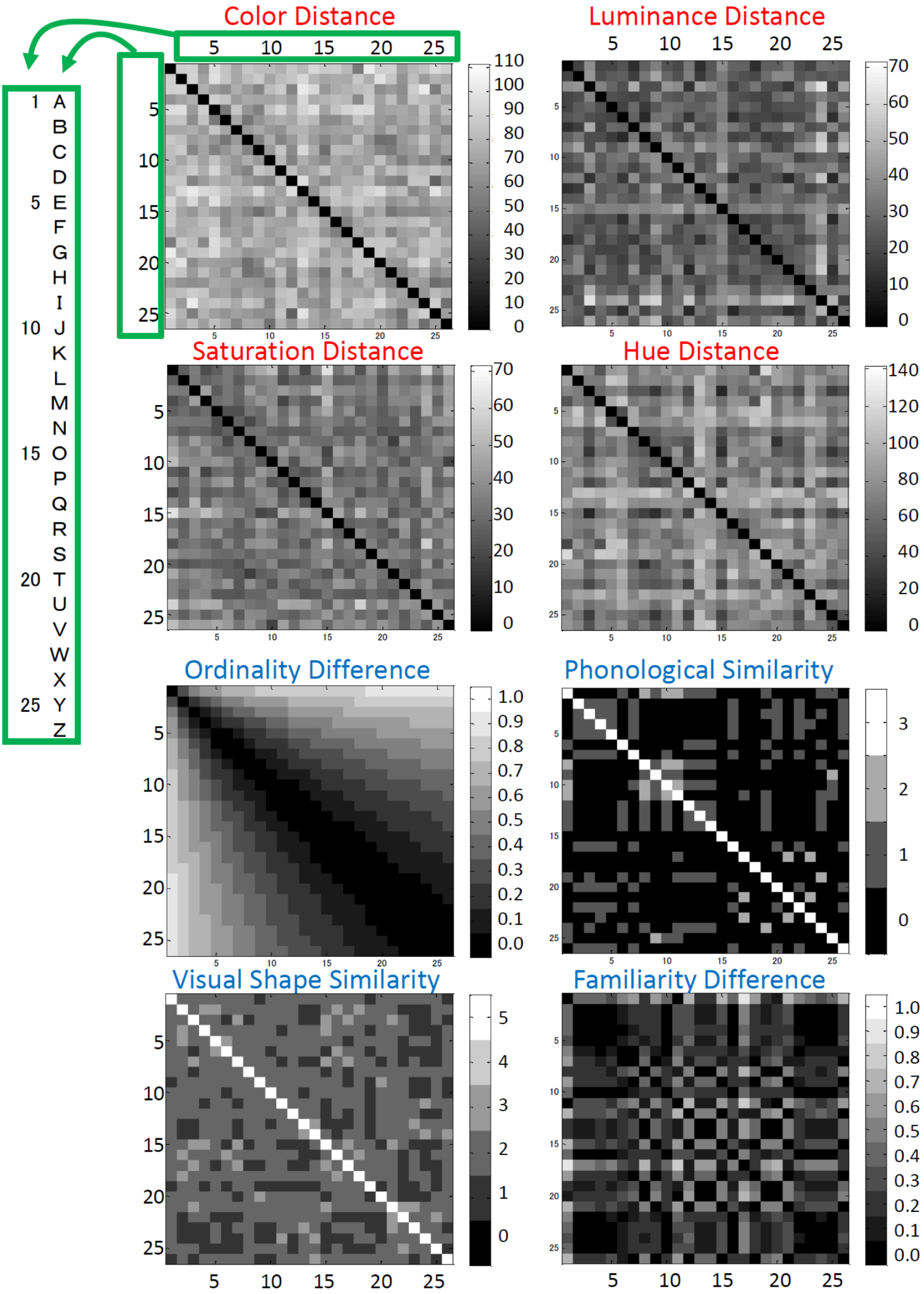

Figure S2. Distance matrices of each color/grapheme property measures for English alphabet letters.
