# Supplementary material for: Grapheme learning and grapheme-color synesthesia: toward a comprehensive model of grapheme-color association
Source: Front Hum Neurosci. 2013 Nov 11;7:757. doi: 10.3389/fnhum.2013.00757 (PMC3822291; doi:10.3389/fnhum.2013.00757)
Supplement: Supplementary file 3 [file DataSheet1.PDF]

Table S1. Order in the sequence, character, Roma-ji, and international phonetic alphabets (IPAs) for Hiragana characters.

44	39	36	31	26	21	16	11	6	1	Order
わ	ら	や	ま	は	な	た	さ	か	あ	Character
WA	RA	YA	MA	HA	NA	TA	SA	KA	A	Roma-ji
ɰa	ɾa	ja	ma	ha	na	ta	sa	ka	a	IPA
	40		32	27	22	17	12	7	2	
	り		み	ひ	に	ち	し	き	い	
	RI		MI	HI	NI	CHI)	SHI	KI	I	
	ɾi		mji	çi	ɲi	tʃi	ʃi	kji	i	
	41	37	33	28	23	18	13	8	3	
	る	ゆ	む	ふ	ぬ	つ	す	く	う	
	RU	YU	MU	FU	NU	TSU	SU	KU	U	
	ɾɯ	jɯ	mɯ	ɸɯ	nɯ	tsɯ̈	sɯ̈	kɯ	ɯ	
	42		34	29	24	19	14	9	4	
	れ		め	へ	ね	て	せ	け	え	
	RE		ME	HE	NE	TE	SE	KE	E	
	ɾe		me	he	ne	te	se	ke	e	
45	43	38	35	30	25	20	15	10	5	
を	ろ	よ	も	ほ	の	と	そ	こ	お	
WO	RO	YO	MO	HO	NO	TO	SO	KO	O	
o/ɰo	ɾo	jo	mo	ho	no	to	so	ko	o	

46
ん
N
ɴ

Notes. This table is created based on a table of IPAs for Japanese phonemes provided by a Japanese dictionary "Daijirin" (3rd edition). See http://daijirin.dual-d.net/extra/nihongoon.html (Japanese only) for the details.
