# Supplementary material for: Grapheme learning and grapheme-color synesthesia: toward a comprehensive model of grapheme-color association
Source: Front Hum Neurosci. 2013 Nov 11;7:757. doi: 10.3389/fnhum.2013.00757 (PMC3822291; doi:10.3389/fnhum.2013.00757)
Supplement: Supplementary file 4 [file DataSheet2.PDF]

Table S2. Correlations among the variables for Hiragana characters when 5 character pairs were included in a bin (N = 207).

	Color dist.	Lum. dist.	Sat. dist.	Hue dist.	Ord. diff.	Phono. sim.	Shape sim.
Color distance							
Luminance distance	0.64**						
Saturation distance	0.83**	0.65**					
Hue distance	0.89**	0.47**	0.61**				
Ordinality difference	0.51**	0.31**	0.42**	0.42**			
Phonological similarity	-0.49**	-0.35**	-0.44**	-0.50**	-0.27**		
Shape similarity	-0.36**	-0.27**	-0.38**	-0.31**	-0.09	0.21**	
Familiarity difference	0.38**	0.11	0.35**	0.31**	0.38**	-0.17*	-0.09

* $p < .05$, ** $p < .01$
