# Supplementary material for: Grapheme learning and grapheme-color synesthesia: toward a comprehensive model of grapheme-color association
Source: Front Hum Neurosci. 2013 Nov 11;7:757. doi: 10.3389/fnhum.2013.00757 (PMC3822291; doi:10.3389/fnhum.2013.00757)
Supplement: Supplementary file 5 [file DataSheet3.PDF]

Table S3. Results of multiple regression analyses for Hiragana character pairs when 5 character pairs were included in a bin.

Variables	Unstandardized Coefficient		Standardized Coefficient	*t*-value	*p*-value
	B	*SE*	β		
Color distance (R^2 = .48, $F(4, 202) = 47.32$, $p < .01$)					
Ordinality difference	27.556	4.568	0.339	6.03	< .01
Phonological similarity	-16.161	2.720	-0.319	-5.94	< .01
Shape similarity	-13.119	2.764	-0.246	-4.75	< .01
Familiarity difference	24.397	7.764	0.173	3.14	< .01
Luminance distance (R^2 = .21, $F(4, 202) = 13.41$, $p < .01$)					
Ordinality difference	7.849	2.232	0.244	3.52	< .01
Phonological similarity	-4.937	1.329	-0.247	-3.71	< .01
Shape similarity	-4.177	1.351	-0.198	-3.09	< .01
Familiarity difference	-2.415	3.794	-0.043	-0.64	.53
Saturation distance (R^2 = .40, $F(4, 202) = 33.70$, $p < .01$)					
Ordinality difference	8.195	2.018	0.246	4.06	< .01
Phonological similarity	-5.860	1.201	-0.282	-4.88	< .01
Shape similarity	-6.201	1.221	-0.284	-5.08	< .01
Familiarity difference	10.714	3.430	0.185	3.12	< .01
Hue distance (R^2 = .39, $F(4, 202) = 32.64$, $p < .01$)					
Ordinality difference	22.837	5.519	0.252	4.14	< .01
Phonological similarity	-20.827	3.286	-0.369	-6.34	< .01
Shape similarity	-11.890	3.340	-0.200	-3.56	< .01
Familiarity difference	20.402	9.381	0.130	2.17	< .05

Note. SE = standard error.
